# Mito-TEMPO Mitigates Fibromyalgia Induced by Reserpine in Rats: Orchestration Between SIRT1, Mitochondrial Dynamics, Endoplasmic Reticulum and miRNA-320

**DOI:** 10.1007/s11064-025-04424-9

**Published:** 2025-05-28

**Authors:** Heba S. Zaky, Nermin T. El-Said, Amany S. Aboutaleb, Albatoul Allam, Mona Mansour, Hebatalla I. Ahmed, Somaia A. Abdel-Sattar

**Affiliations:** Pharmacology and Toxicology Department, Faculty of Pharmacy for Girls, Al-Azhar University, Nasr City, Cairo P.N.11754, Egypt

**Keywords:** Fibromyalgia, Mito-TEMPO, SIRT 1, miRNA, Mitochondrial dysfunction, Rats

## Abstract

Fibromyalgia (FM) is a chronic disorder that lacks both well-defined underlying causes and effective treatments. Mito-TEMPO (MIT) is a mitochondrial-specific antioxidant that has demonstrated benefits in many cancerous, renal, cardiovascular, and neurodegenerative disorders. However, the therapeutic effect of MIT on FM remains ambiguous. The objective of the current work is to illuminate the use of MIT for FM and its prospective mechanisms. Here, we used the FM rat model induced by three days of subcutaneous reserpine injection (1 mg/kg) and examined the role of MIT on SIRT1 activation and other implicated molecular pathways. Behavioral tests showed that MIT (0.7 mg/kg) can effectively alleviate the locomotor, nociceptive, and depressive-like behaviors in reserpinized rats, an effect that simultaneously reconciles the balance of monoamines in the rat brain. Western blot analysis showed that MIT up-regulates SIRT1 and improves the expression of mitochondrial dynamics proteins (DRP1 and OPA1) and the endoplasmic reticulum protein (CHOP). Furthermore, MIT treatment significantly enhanced the SOD and CAT activities and decreased the brain contents of NF-κB, TNF-α, and BAX, but significantly enriching the Bcl-2 content. Lastly, MIT treatment significantly reduced the genetic expression of miRNA-320 following RES treatment. All the measured parameters showed a significant correlation with SIRT1 expression. Our results suggest that MIT provides antioxidant, anti-apoptotic, and anti-inflammatory impacts on the FM rat model, with proposed mechanisms involved activating the SIRT1 pathway to regulate mitochondrial dynamics, endoplasmic reticulum stress, as well as miRNA-320. Thus, MIT has the potential to be an effectual drug candidate for FM treatment.

**Supplementary Information:**

The online version contains supplementary material available at 10.1007/s11064-025-04424-9.

## Introduction

Fibromyalgia (FM) is an increasingly prevalent pain syndrome. It is usually presented with stiff joints, fatigue, cognitive dysfunctions, depressive symptoms, and sleep disturbances. The importance of research in FM is growing due to its significant influence on patients’ daily life and the economic strain that is imposed on healthcare systems [1]. The underlying causes of FM are not well understood, but it is believed that genetic predispositions, environmental factors, and neuromodulator processes may all play roles in the emergence and progression of the condition [2].

One well-recognized theory about the origins of FM involves the alteration of both central and peripheral processing of pain; an upsurge in the pro-nociceptive ascending trajectory signaling, and a diminution in that of the descending (anti-nociceptive) pathways [3]. This abnormal pain processing is linked to FM patients’ heightened sensitivity to thermal and other painful stimuli [3]. At the molecular levels, multiple interactive mechanisms can underlie altered pain processing in FM, namely mitochondrial impairment, endoplasmic reticulum (ER) stress modulation, oxidative stress modulation, and inflammatory and immune processes dysregulation [4–6].

A growing body of research suggests a reciprocal linkage between dysfunctional mitochondria and the upsurge of oxidative stress. Diminished mitochondrial functionality can reduce the cell’s free radicals neutralization ability, leading to reactive oxygen species (ROS) accumulation, which enhances mitochondrial injury and the release of mitochondrial ROS, thus intensifying oxidative stress [7]. Mitochondria-elaborated ROS are reported to play a role in muscular pain by reducing adenosine triphosphate (ATP) availability both in muscular and neural cells [8]. In particular, superoxide free radicals have been identified as principal players in exaggerated central and peripheral nociception in rats [9].

In addition, mitochondrial ROS could trigger the unfolded protein response, and the unfolded protein response could be an upstream of ROS which means mutual interdependence between ROS production and the unfolded protein response or an ER stress is present [10, 11]. The transcription factor C/EBP homologous protein, or CHOP, is a critical regulator of the cellular response to ER stress that can induce cell death if the stress is prolonged or intense [12, 13]. Notably, the reported changes in the ER stress biomarker CHOP in a reserpine (RES)-induced FM rat model underscore the role of ER stress in FM’s pathophysiology [14].

Furthermore, the production of proinflammatory cytokines, usually associated with neuronal inflammation and FM pain syndromes, may be exacerbated by mitochondrial dysfunction and oxidative stress [15, 16]. As well, the neurogenic inflammation, developed by the C fibers-mediated release of inflammatory neuropeptides, also plays a significant role in FM, contributing to allodynia and other forms of pain sensitization [17]. Consequently, FM treatments often employ agents that target mitochondrial biogenesis and have anti-oxidative and anti-inflammatory attributes [2].

Besides, promising outcomes from animal experiments suggest the therapeutic potential of microRNAs (miRNAs) in mitigating pain [18]. MicroRNAs are single-stranded RNAs that can affect how different neurotransmitters, ion channels, signaling molecules, and fundamental proteins are expressed, hence, influencing excitability in nociceptive neurons [19–21]. Indeed, the expression profiles of different miRNAs in FM patients are altered from those in healthy individuals [21, 22].

Silent information regulator 1 (SIRT1) is a highly conserved NAD-dependent histone deacetylase that is expressed in various cells and participates in modifying many biological activities, including mitochondrial biogenesis, neurogenesis, cellular responses to stressful conditions, and apoptosis [23–27]. Indeed, activated SIRT1-PGC1α signaling could potentially improve dynamin-related protein 1 (DRP1)-mediated mitochondrial fission and suppressed mitochondrial ROS production and cellular apoptosis in diabetic mice [28]. Similarly, activation of SIRT1 could inhibit the PERK-eIF2α-CHOP axis of the ER stress response in tert-Butyl hydroperoxide-treated rat chondrocytes [29]. The anti-inflammatory effects of SIRT1 have been also demonstrated and suggested to be mediated via inhibiting various expression factors involved in inflammatory pathways such as NF-kB, HIF1a, and P38MAPK [30].

Under normal circumstances, SIRT1 exists in the nucleus and the cytoplasm where it deacetylates multiple non-histone targets namely nuclear factor-kappa B (NF-κB), p53, FOXO3, and others [31]. Emerging studies have demonstrated a negative correlation between SIRT1 and pain perception in patients suffering from enduring pain [32–34]. At the molecular level, SIRT1 activation revealed positive outcomes in chronic pain via regulating mitochondrial dysfunction, oxidative stress, and inflammation [35, 36]. Therefore, SIRT1 activators may serve as potential therapeutics for FM pain management.

Mito-TEMPO (MIT) is a mitochondrial superoxide and free radical scavenger that enters and accumulates in the mitochondrial matrix with the aid of the positive charge of the triphenylphosphonium moiety [37]. By ameliorating mitochondrial dysfunction and diminishing oxidative stress, MIT has demonstrated benefits in many pathologic conditions namely cancerous, neurodegenerative, renal and cardiovascular disorders [38–46]. Worth mentioning, MIT was previously explored as a neuropathic pain reliever in an experimental model of chronic nerve constrictive injury [47]. However, its effect on FM pain management has not yet been explored. In the current study, the beneficial outcomes of MIT against the RES-induced FM model in rats were examined at the behavioral and molecular levels. Our strategy centers on whether the activation of SIRT1 by MIT could be implemented in ameliorating FM via the regulation of various molecular pathways, including mitochondrial dynamics, oxidative stress, ER stress, microRNA regulation, and inflammatory and apoptotic processes.

## Materials and Methods

### Chemicals and Reagents

Mito-TEMPO (MIT), RES, pregabalin (PG), and all other chemical substances were purchased from Sigma-Aldrich, St. Louis, MO. MIT, RES, and PG were dissolved in the corresponding vehicle (physiological saline, 0.5% glacial acetic acid in distilled H_2_O, and distilled H_2_O, respectively) to the required doses and administered to animals by the appropriate routes.

### Animal Grouping and Drug Administration

At the Faculty of Pharmacy for Girls, Al-Azhar University, twenty-four male Sprague-Dawley rats (Nile Company for Drugs, Cairo, Egypt) weighing between 150 and 180 g were accommodated in a 12-hour light/dark cycle, with a standard diet and unlimited access to water. The rats were cared for at a temperature of 25 ± 2 C and a humidity of 60–70%. The Ethics Committee of Al-Azhar University’s Faculty of Pharmacy for Girls permitted all of the experiments (455/2024), which were carried out in compliance with the NIH Guide for the Principles of Laboratory Animal Care [85 − 23/2011].

Following a one-week period of adaptation, rats were randomly allocated into four groups; Vehicle control (VEH) group was injected subcutaneously (s.c.) with 1 mL/kg of the vehicle once a day for three days, then injected intraperitoneal (i.p.) with saline injection (1 mL/kg) for 14 days; Fibromyalgia model (RES) group was administered s.c. RES (1 mg/kg) for a period of three days [48], tailed by intraperitoneal (i.p.) saline injection as in group (VEH); Pregabalin-treated RES group (RES + PG) was treated with RES as in (RES) group, followed by oral PG (30 mg/kg) single dose every day for a period of 14 days consecutively [49]; Mito-TEMPO treated RES group (RES + MIT) was treated with RES as in (RES) group, followed by MIT (0.7 mg/kg, i.p., once a day) for a period of 14 days [47].

Then, behavioral tests were conducted sequentially; on day 18, the open field test (OFT) was performed, followed by the hot plate test on day 19, and finally, on day 20, the forced swimming test (FST) was conducted. Rats were euthanized on the twenty-first day of the experiment, and their brains were immediately removed and placed in liquid nitrogen for subsequent biochemical, western blotting, or real-time quantitative polymerase chain reaction (RT-qPCR) scrutinization.

### Behavioral Assessments

#### Open Field Test

The OFT is frequently used to appraise rodents’ locomotor and exploratory behaviors. The tool used is an 80 × 80 × 40 cm box made of wood with a white floor partitioned into 16 similar squares and red inner edges. A period of three minutes was used to appraise the latency time to start moving, the grooming behavior and the ambulation and rearing frequencies [50, 51].

#### Hot Plate Test

Thermal nociception is commonly assessed in rodents using the hot plate test. The animal is restrained onto a hot plate regulated at a temperature of 55 ± 1 C. The latency or how long it took a rat to jump out or lick its hind paw was measured. As a precaution, a one-minute maximum delay time was set to avoid any bodily harm [52].

#### Forced Swimming Test

The FST is frequently used to assess rodents’ depressive-like behavior. The animal is forced to swim in a transparent plexiglass cylinder (20 cm diameter × 50 cm height) filled with water to a depth of 30 cm. No physical support with paws or tails can be attained at that depth. Active (swimming or climbing with the front paws shatter the water surface by rapid movements) and passive (immobility with no movement) behaviors were recorded in a period of five minutes manually. The immobility behavior is seen to be a sign of acknowledged helplessness, which is typically connected to severe depression [53, 54].

### Colorimetric Assessment of Antioxidant Enzymes

Using the proper kits purchased from Biodiagnostic Co., Cairo, Egypt., the antioxidant enzymes catalase (CAT) and superoxide dismutase (SOD) activities were assessed (catalog no.: SD 2521 and CA 2517, respectively).

### ELISA Assay for Biogenic Amines and Inflammatory and Apoptotic Biomarkers

Levels of biogenic amines; 5-hydroxytryptamine (5-HT), dopamine (DA), and norepinephrine (NE), and inflammatory response biomarkers; TNF-α, and NF-κB, were assessed in brain tissue using the respective rat-specific Enzyme-linked immunosorbent assay (ELISA) kits (catalog no.: MBS166089, MBS725908, MBS269993, MBS825075, and MBS453975, respectively) supplied by MyBiosource, San Diego, USA. Also, Bcl-2-associated X protein (BAX) and B cell lymphoma-2 (Bcl-2) ELISA kits were procured from Cusabio Biotech Co., Wuhan, China (catalog no.: CSB-EL002573RA, and CSB-E08854r, respectively), and utilized for assessing relevant proteins. All tests were carried out according to the instructions provided by the manufacturer.

### Western Blot Assessment

Western blot was used to approximate the relative expression of dynamin-related protein 1 (DRP1), optic atrophy factor 1 (OPA1), CHOP, and SIRT1, as previously expressed by Zaky and coworkers. Following extraction by the lysis buffer, RIBA (Santa Cruz, CA, USA), total proteins were estimated utilizing the Bradford Protein Assay Kit (catalog no.: 23200). Then, denaturation of equivalent amounts of proteins was done using 2x Laemmli sample buffer, resolved using sodium dodecyl sulfate-polyacrylamide gel electrophoresis, and blotted on membranes containing polyvinylidene fluoride. TBS-Tween 20 was used to block the membranes and they were probed for a night with the primary antibodies against DRP1 (1:1000, catalog no.: MA5-38045), OPA1 (1:1000, catalog no.: PA5-98029), CHOP (1:1000, catalog no.: PA5-88116), and SIRT1 (dilution 1:1000, catalog no.: MA5-27217). Probing with β-actin antibody (dilution 1:1000, catalog no.: MA5-32540) was done to certify equal loading of proteins. The membranes were washed and incubated with HRP-conjugated anti-goat IgG antibody (dilution 1:4000, catalog no.: A-11006) at room temperature for 1 h. The supplier for all antibodies used was Thermofischer Scientific, which is located in MA, USA. In order to detect the signals, an enhanced chemiluminescence kit was purchased from Beyotime in Shanghai, China. The manufacturer’s instructions were precisely followed [55].

### Gene Expression Assessment Using RT-qPCR

RT-qPCR was used to determine the relative expression of miRNA-320 in brain tissues. According to Aboutaleb et al. brief description, the mirVana miRNA Isolation Kit (catalog no.: AM1560) was purchased from Thermofisher Scientific, MA, USA, and used for total RNA (including miRNAs) extraction. Then, total RNA was reverse transcribed using TaqMan miRNA Reverse Transcription Kit (catalog no.: 4366596), as directed by the manufacturer Thermofisher Scientific, MA, USA. The sequence-specific primer for miRNA-320; F: 5’-GGGGGAAAGCTGGGTTG-3′ and R: 5’-GTGCGTGTCGTGGAGTCG-3′ was used to amplify cDNA samples in real-time PCR. All reactions were triplicated and normalized to the housekeeping gene U6 snRNA; F: 5′-CTCGCTTCGGCAGCACA-3′ and R: 5′-AACGCTTCACGAATTTGCGT-3′ and the relative expressions were quantified using the 2^−ΔΔCT^ method [56].

### Data Analysis

Version 5 of GraphPad Prism software (San Diego, USA) was used for statistical analysis and graph sketching. The variable values were expressed as means $\:\pm\:$ standard deviation (SD). Means of different groups were compared using One-way analysis of variance (ANOVA) with Tukey’s post hoc test. The P values less than 0.05 were considered significant. Meanwhile, the correlation between some variables was assessed using Pearson’s correlation test, considering those with a moderate (0.4 ≤ *r* < 0.7) or strong (*r* ≥ 0.7) correlation degree and P values below 0.05 are statistically significant [57].

## Results

### Mito-TEMPO Modulates the RES-Induced Brain Monoamines Imbalance in Rats

**Fig. 1:**
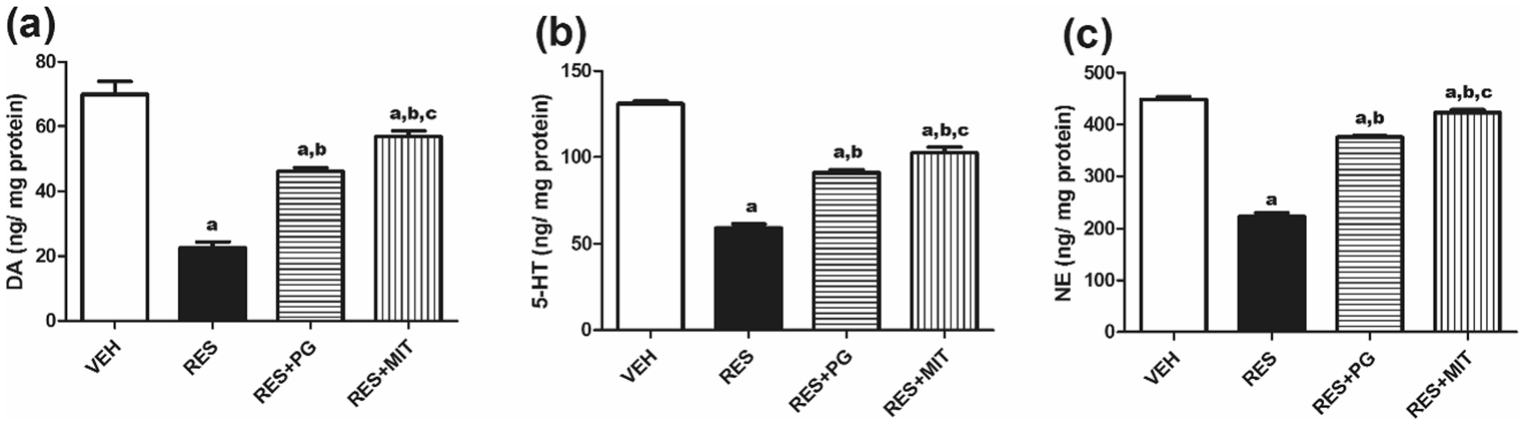
Effect of Mito-TEMPO on fibromyalgia-induced changes in biogenic amines in rats’ brain. Values explicate the mean ± SD (*n* = 6). Statistics were carried out by ANOVA tailed by Tukey’s as post-hoc test. **a**, **b**, **c** statistically significant from VEH, RES and RES + PG groups respectively at *p* < 0.05. ANOVA: analysis of variance; 5-HT: 5-hydroxytryptamine; VEH: Vehicle group; DA: Dopamine; RES: Fibromyalgia model group; RES + MIT: Mito-TEMPO-treated fibromyalgia group; RES + PG: pregabalin-treated fibromyalgia group; NE: Norepinephrine; SD: Standard deviation

Figure 1 depicts that RES administration significantly decreased the brain content of DA, 5-HT and NE to 32.4, 45.3, and 49.6% of the VEH group. MIT, given to the reserpinized rats, markedly boosted the brain content of DA, 5-HT and NE reaching 2.5, 1.5, and 1.9 folds, respectively, of the non-treated reserpinized rats’ values. Of note, MIT prioritized PG in rectifying the DA, 5-HT and NE brain contents in the reserpinized rats.

### Mito-TEMPO Diminishes RES-Induced Behavioral Changes in Rats

**Fig. 2:**
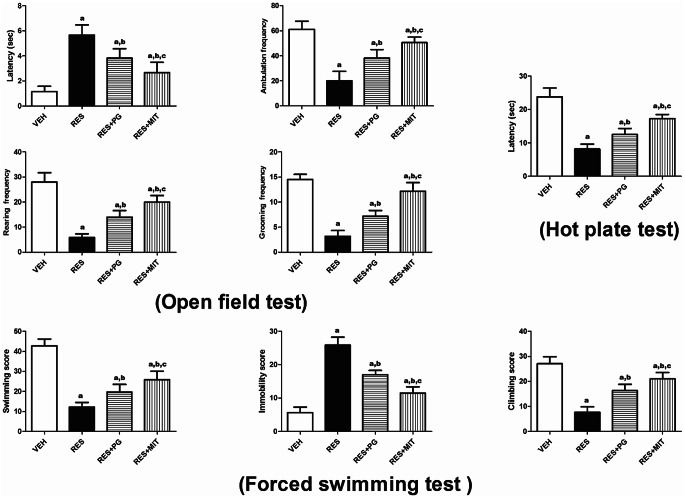
Effect of Mito-TEMPO on fibromyalgia-induced behavioral changes in rats. Values explicate the mean ± SD (*n* = 6). Statistics were carried out by ANOVA tailed by Tukey’s as post-hoc test. **a**, **b**, **c** statistically significant from VEH, RES and RES + PG groups respectively at *p* < 0.05. ANOVA: analysis of variance; VEH: Vehicle group; RES: Fibromyalgia model group; RES + MIT: Mito-TEMPO -treated fibromyalgia group; RES + PG: Pregabalin-treated fibromyalgia group; no: Number; SD: Standard deviation; sec: Seconds

In the OFT, RES noticeably diminished both locomotor and exploratory behaviors presented as an increase in the latency by 3.8 folds and a diminution in ambulation, rearing and grooming frequencies by 67.3, 79.3 and 77.9%, respectively, in contrast against the VEH group. In addition, it brought a marked nociceptive sensitivity appeared as a reduction in the latency time in the hot plate test by 65.5% relative to the VEH group. No doubt RES also induced a depressive-like behavior that revealed in the FST as significantly increased immobility score by 3.5 folds and decreased both swimming and climbing scores by 71.5%, with respect to the VEH-treated rats. MIT administration to reserpinized rats significantly reversed these behavioral changes as it decreased the latency by 52.6% and induced a 1.5-, 2.4-, and 2.8-fold upsurge in the ambulation, rearing, and grooming frequencies, respectively, in OFT with respect to non-treated animals. Additionally, MIT increased the latency time in the hot plate assessment to 2.1 folds with respect to reserpinized rats. Finally, MIT decreased the score of immobility by 55.4% and induced a 1.1- and 1.7- fold rise in the scores of swimming and climbing, respectively, in FST with respect to reserpinized rats. Compared to PG, MIT revealed a noticeable enhancement in all behavioral parameters tested (Fig. 2).

### Mito-TEMPO Ameliorates the Brain Oxidative Stress Induced by RES in Rats’ Brain

Data of Table 1 unveiled that administration of RES increased the brain oxidative stress presented as a significant diminishing of both SOD and CAT activities by 61.9% and 71.1%, respectively, compared to VEH group. Inversely, MIT treatment of reserpinized rats remarkably enhanced the brain activities of SOD and CAT to 2.1 and 3 folds, respectively, relative to the untreated-reserpinized rats with a more remarkable effect of MIT than PG.

**Table 1:** Effect of Mito-TEMPO on fibromyalgia-induced oxidative stress in rats’ brain

	VEH Mean ± SD	RES Mean ± SD	RES + PG Mean ± SD	RES + MIT Mean ± SD
SOD (U/mg protein)	87.7 ± 5.9	33.4 ± 6.4^a^	59.3 ± 5.0^a, b^	71.6 ± 4.7^a, b, c^
CAT (U/mg protein)	204.6 ± 6.6	59.2 ± 13.2^a^	138.4 ± 4.1^a, b^	174.7 ± 6.2^a, b, c^

### Mito-TEMPO Mitigates RES-induced Changes in Mitochondrial Dynamics in Rats’ Brain

**Fig. 3:**
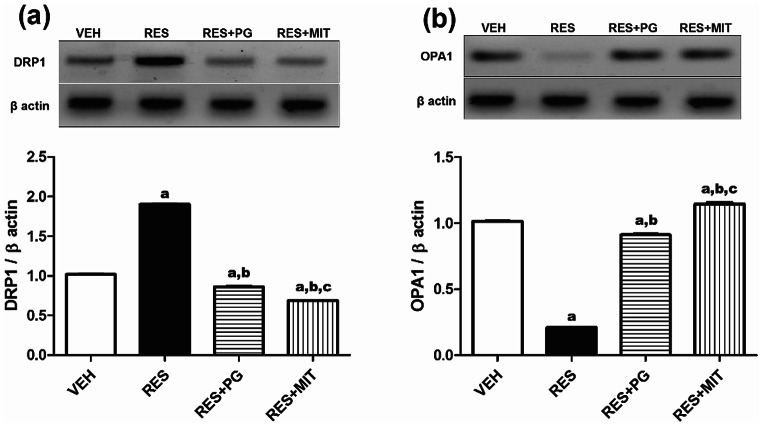
Effect of Mito-TEMPO on DRP1 and OPA1 protein expression changes in reserpinized rats’ brain. Values explicate the mean ± SD (*n* = 6). Statistics were carried out by ANOVA tailed by Tukey’s as post-hoc test. **a**, **b**, **c** statistically significant from VEH, RES and RES + PG groups respectively at *p* < 0.05. ANOVA: analysis of variance; VEH: Vehicle group; DRP1: Dynamin-related protein 1; RES: Fibromyalgia model group; RES + MIT: Mito-TEMPO-treated fibromyalgia group; RES + PG: Pregabalin-treated fibromyalgia group; OPA1: Optic atrophy 1; SD: Standard deviation

Results of the current work show a noticeable upsurge in DRP1 expression and a marked lessening in OPA1 by 86.9% and 79.2%, respectively in RES-treated animals, with respect to the VEH group. Dysregulated mitochondrial dynamic significantly improved by administration of MIT to reserpinized animals revealed as a significant decrease in DRP1 expression, while significantly increased OPA1 expression in rats’ brain by 63.8% and 4.4 folds, respectively, with respect to the RES group. Again, MIT effect surpasses that of PG (Fig. 3a, b).

### Mito-TEMPO Modulates RES-induced CHOP Expression in Rats’ Brain

**Fig. 4:**
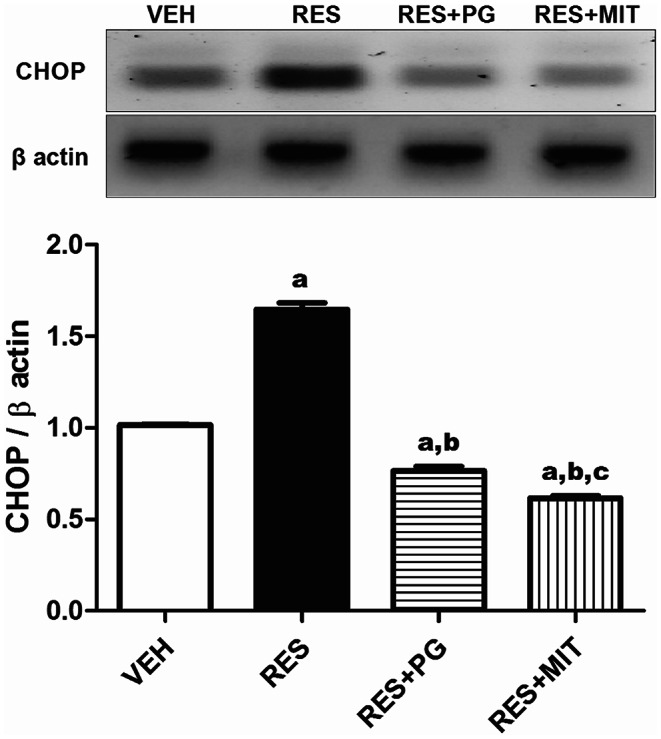
Effect of Mito-TEMPO on CHOP protein expression changes in reserpinized rats’ brain. Values explicate the mean ± SD (*n* = 6). Statistics were carried out by ANOVA tailed by Tukey’s as post-hoc test. **a**, **b**, **c** statistically significant from VEH, RES and RES + PG groups respectively at *p* < 0.05. ANOVA: analysis of variance; VEH: Vehicle group; CHOP: C/EBP homologous protein; RES: Fibromyalgia model group; RES + MIT: Mito-TEMPO-treated fibromyalgia group; RES + PG: Pregabalin-treated fibromyalgia group; SD: Standard deviation

The results demonstrated in Fig. 4 emphasize the role of RES to induce endoplasmic reticulum stress, which showed as a significant increase in CHOP protein expression reaching 1.6 folds of the VEH group. MIT given to the reserpinized rats diminished CHOP protein expression to 37.4% of the non-treated reserpinized rats. MIT results induced a more significant CHOP modulation than PG.

### Mito-TEMPO Alleviates RES-induced SIRT1 Expression Alterations in Rats’ Brain

**Fig. 5:**
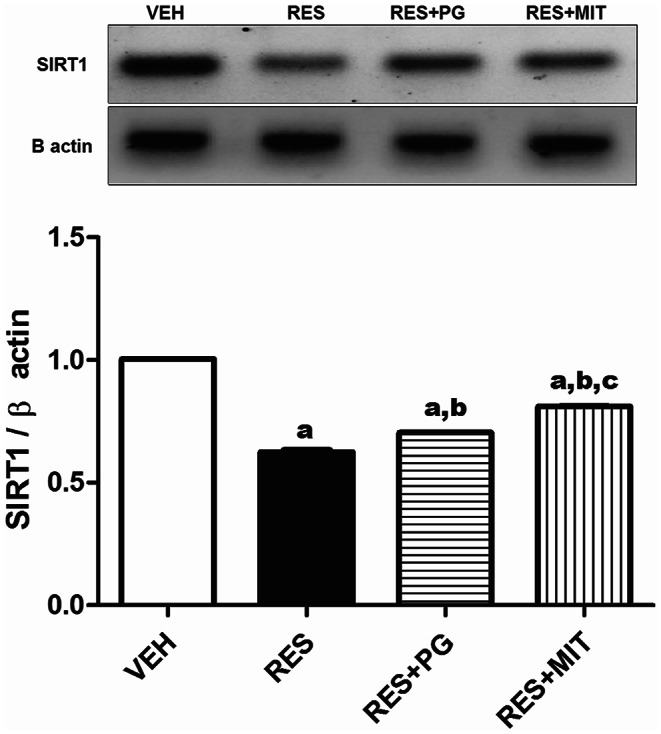
Effect of Mito-TEMPO on SIRT1 protein expression changes in reserpinized rats’ brain. Values explicate the mean ± SD (*n* = 6). Statistics were carried out by ANOVA tailed by Tukey’s as post-hoc test. **a**, **b**, **c** statistically significant from VEH, RES and RES + PG groups respectively at *p* < 0.05. ANOVA: analysis of variance; VEH: Vehicle group; RES: Fibromyalgia model group; RES + MIT: Mito-TEMPO-treated fibromyalgia group; RES + PG: Pregabalin-treated fibromyalgia group; SD: Standard deviation; SIRT1: Silent information regulator 1

The present work results demonstrate a significant reduction in brain SIRT1 protein expression by RES administration, reaching 62.3% of the VEH group. MIT injected to reserpinized rats significantly increased the SIRT1 protein expression of the brain to 1.3 fold compared to the reserpinized rats. Notably, SIRT1 modulation is more marked with MIT than PG (Fig. 5).

### Mito-TEMPO Ameliorates the Inflammatory Response Induced by RES in Rats’ Brain

**Fig. 6:**
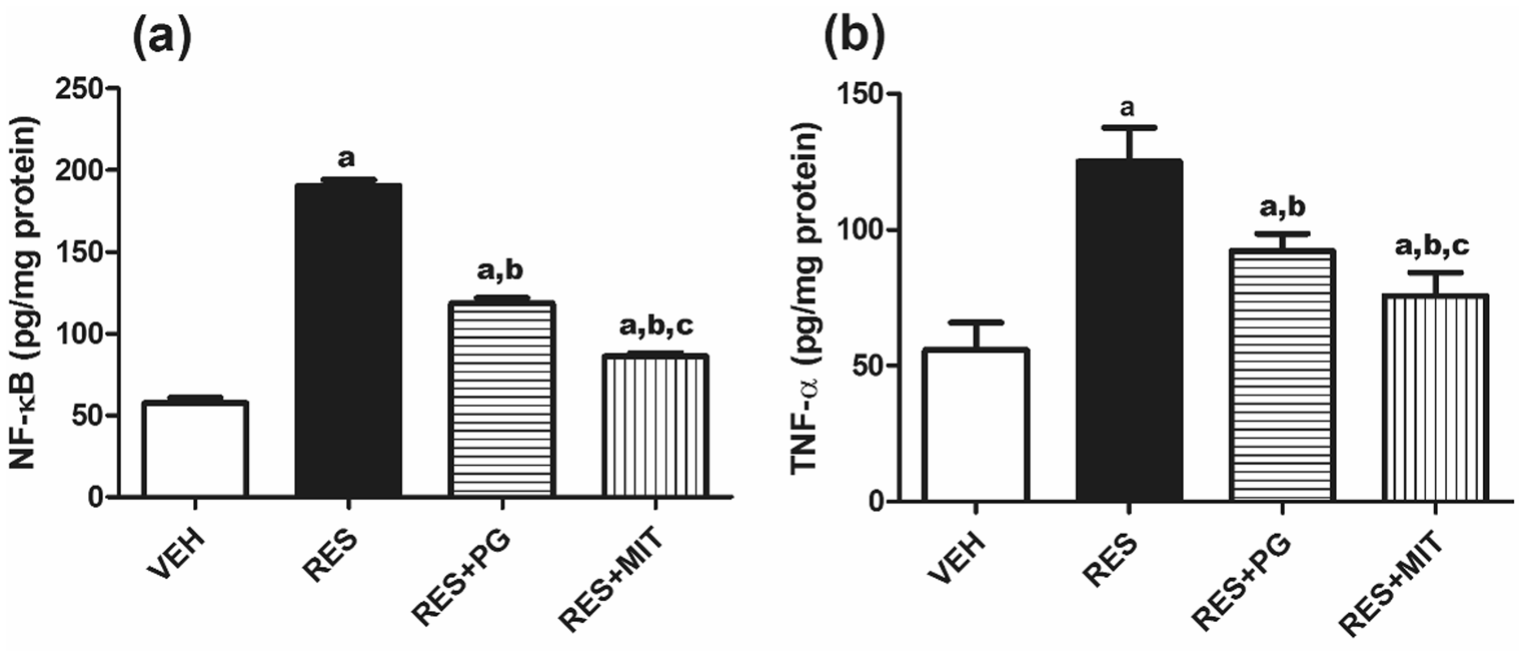
Effect of Mito-TEMPO on changes in NF-κB and TNF-α content in reserpinized rats’ brain. Values explicate the mean ± SD (*n* = 6). Statistics were carried out by ANOVA tailed by Tukey’s as post-hoc test. **a**, **b**, **c** statistically significant from VEH, RES and RES + PG groups respectively at *p* < 0.05. ANOVA: analysis of variance; VEH: Vehicle group; RES: Fibromyalgia model group; RES + MIT: Mito-TEMPO-treated fibromyalgia group; RES + PG: Pregabalin-treated fibromyalgia group; NF-κB: Nuclear factor-kappa B; SD: Standard deviation; TNF-α: Tumour necrosis factor alpha

As shown in Fig. 6a and b, RES induced a marked upsurge in the NF-κB and TNF-α brain content, reaching 3.3 folds and 2.2 folds, respectively, compared to the VEH-treated rats. MIT treatment of rserpinized animals significantly reduced NF-κB and TNF-α to 45.2% and 60.5%, respectively, with respect to non-treated reserpinized rats. The anti-inflammatory effect is more noticeable with MIT than with PG.

### Mito-TEMPO Alleviates Apoptotic Changes Induced by RES in Rats’ Brain

**Fig. 7:**
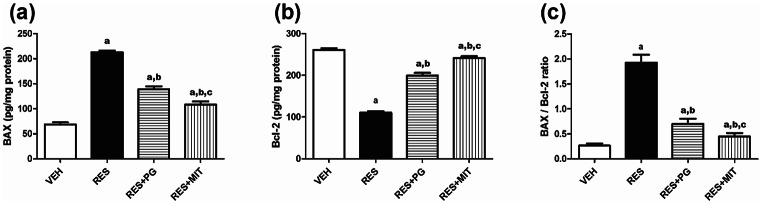
Effect of Mito-TEMPO on changes in BAX and Bcl-2 content in reserpinized rats’ brain. Values explicate the mean ± SD (*n* = 6). Statistics were carried out by ANOVA tailed by Tukey’s as post-hoc test. **a**, **b**, **c** statistically significant from VEH, RES and RES + PG groups respectively at *p* < 0.05. ANOVA: analysis of variance; BAX: Bcl-2-associated X protein; Bcl-2: B-cell lymphoma; VEH: Vehicle group; RES: Fibromyalgia model group; RES + MIT: Mito-TEMPO-treated fibromyalgia group; RES + PG: Pregabalin-treated fibromyalgia group; SD: Standard deviation

Figure 7a, b demonstrates a significant 3.1-fold increase in BAX and a significant decrease in Bcl-2 by 57.6% following RES administration, relative to the VEH-treated rats. MIT treatment markedly lessened BAX and increased Bcl-2 by 49% and 118.3%, respectively relative to the RES group. Subsequently, the intrinsic mitochondrial apoptotic indicator BAX/ Bcl-2 ratio was significantly elevated in the reserpinized rats up to 7.3 folds of the VEH group, while administration of MIT noticeably reduced that ratio to 23%, relative to the untreated reserpinized animals (Fig. 7c). Current results also elucidate that MIT prioritized PG in modulating these brain apoptotic changes.

### Mito-TEMPO Modulates Brain miRNA-320 Expressions in RES-Treated Rats

**Fig. 8:**
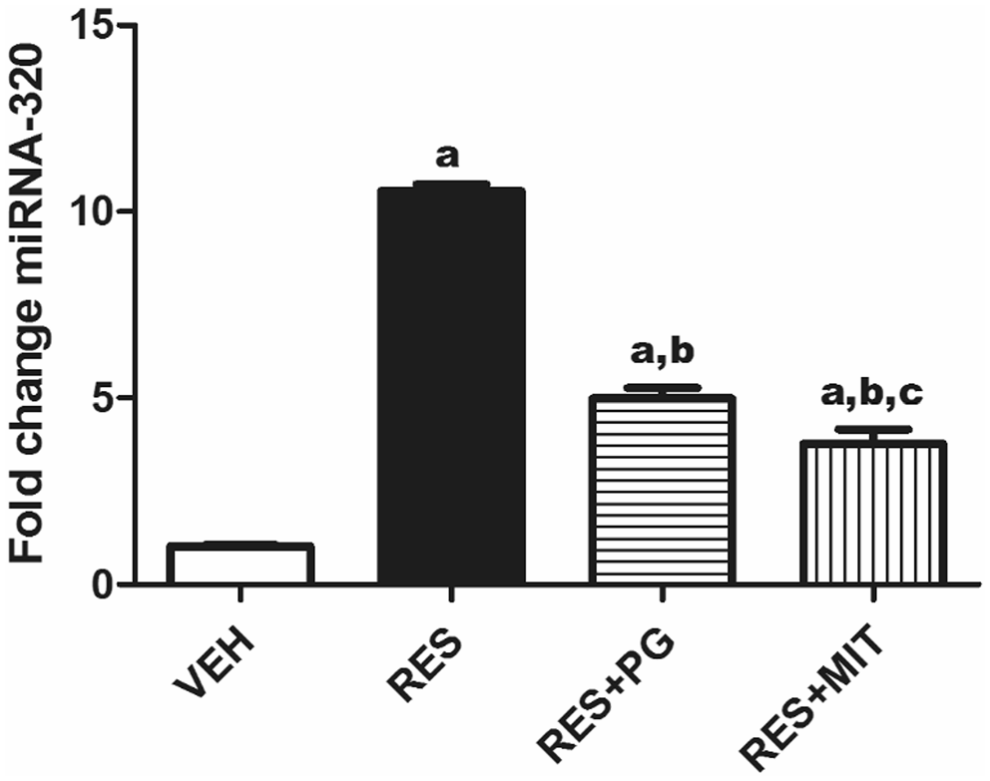
Effect of Mito-TEMPO on changes in miRNA-320 gene expression in reserpinized rats’ brain. Values explicate the mean ± SD (*n* = 6). Statistics were carried out by ANOVA tailed by Tukey’s as post-hoc test. **a**, **b**, **c** statistically significant from VEH, RES and RES + PG groups respectively at *p* < 0.05. ANOVA: analysis of variance; VEH: Vehicle group; RES: Fibromyalgia model group; RES + MIT: Mito-TEMPO-treated fibromyalgia group; RES + PG: Pregabalin-treated fibromyalgia group; miRNA-320: Micro RNA-320; SD: Standard deviation

In RES-administered rats, the brain expression of miRNA-320 exhibited a 10.2-fold increase when compared to the VEH-treated animals. Reserpinized rats injected with MIT exhibited a significant diminish in the brain gene expression of miRNA-320 to 35.9% of reserpinized rats’ results (Fig. 8). Yet, MIT surpassed PG in decreasing the gene expression of miRNA-320 in the reserpinized rats’ brain.

### Correlation Study

Table 2 denotes the Pearson’s correlation coefficients obtained between SIRT1 protein expression and different investigated parameters. Pearson’s r correlation coefficients revealed a positive association of SIRT1 with OPA1 protein expression, CAT, SOD, and Bcl-2. However, SIRT1 was associated negatively with DRP1 protein expression, CHOP protein expression, NF-κB, TNF-α, BAX, and miRNA-320 expression.

**Table 2:** Pearson’s coefficients between SIRT1 expression and DRP1, OPA1, CHOP, CAT, SOD, NF-κB, TNF-α, BAX, Bcl-2, and miRNA-320

Parameter	*r*	*p*
DRP1 (protein expression)	0.5160 ^**b**^	0.0099
OPA1 (protein expression)	0.6830 ^**a**^	0.0002
CHOP (protein expression)	0.4097 ^**b**^	0.0468
CAT (U/mg protein)	0.9042 ^**a**^	< 0.0001
SOD (U/mg protein)	0.9268 ^**a**^	< 0.0001
NF-κB (pg/mg protein)	0.9069 ^**b**^	< 0.0001
TNF-α (pg/mg protein)	0.9080 ^**b**^	< 0.0001
BAX (pg/mg protein)	0.9168 ^**b**^	< 0.0001
Bcl-2 (pg/mg protein)	0.8652 ^**a**^	< 0.0001
miRNA-320 (gene expression)	0.9061 ^**b**^	< 0.0001

a: Statistically significant positive correlation at *p* < 0.05

b: Statistically significant negative correlation at *p* < 0.05

## Discussion

In the current study, we have demonstrated for the first time that the mitochondrial-specific antioxidant MIT intervention in a RES rat model of FM alleviates pain and reduces the associated depressive symptoms, an effect that remarkably surpasses that of PG, a standard treatment approved by the FDA for FM management [49], and is anticipated to be exerted by SIRT1 activation and subsequently alteration of mitochondrial dynamics, microRNA expression, endoplasmic reticulum stress, oxidative stress, and inflammation.

Reserpine is widely used in research to induce symptoms like those seen with FM in human. By depleting biogenic amines, RES has been observed to cause musculoskeletal issues, mirroring the characteristics of FM [58]. There is no doubt that DA, NE, and 5-HT play a vital part in conserving different homeostatic functions, including cognition, memory, mood, and sleep and pain regulation [59, 60]. In particular, NE and 5-HT are the principal biogenic amines mediating the descending inhibitory pathway, whereas dysfunction in the mesolimbic dopaminergic pathway is linked to aggravating nociceptive stimuli perception [61, 62].

In this research, administering MIT to FM model rats led to elevations in brain levels of biogenic amines, which are directly linked to improving locomotion, pain perception, and depressive-like behaviors demonstrated in OFT, hot plate, and FST, respectively. This is consistent with findings reported that MIT considerably modulates central monoaminergic neurotransmission and reduces pain perception and depressive-like behaviors via improving mitochondrial functionality [47, 63].

It is well known that proper mitochondrial functionality is critical for neurons to maintain normal excitability, plasticity, and synaptic transmission [64]. Mitochondria are highly dynamic structures that continuously alter their structure through the processes of fission and fusion. Fission is facilitated by cytosolic dynamin proteins like DRP1, while fusion involves the coordination of mitofusins on the outer membrane, and OPA1 on the inner membrane [65]. Extensive research has explored the implication of mitochondrial dysfunction and oxidative stress in both peripheral and central sensitization, which clarifies the prevalence of chronic pain in FM patients [5]. Furthermore, the connection between mitochondria, oxidative stress, and ER stress is verified, where oxidative stress can trigger ER stress and vice versa, provoking an increased amount of ROS [10, 11].

By linking the deacetylation of multiple substrates to the splitting of NAD^+^, a key indicator of cellular metabolic function, SIRT1 could function as a master metabolic regulator across various tissues [66] with mutual regulation has been demonstrated between SIRT1 and different signaling pathways [30, 55, 56]. Alterations in SIRT1 activity and expression were revealed in numerous metabolic, cardiovascular, neurodegenerative, and cancerous diseases [67]. Regarding pain, SIRT1 up-regulation has revealed positive consequences in chronic pain via regulating oxidative stress, mitochondrial dysfunction, and inflammation [6, 36].

In this investigation, treatment of RES-treated animals with MIT was found to increase OPA-1 expression, while decreasing DRP1 and CHOP expression in brain tissue. These results highlight the interactive communication between mitochondria and the ER [68–70], and align with earlier MIT studies [69, 71–73]. The concurrently enhanced SIRT1 expression in reserpinized rats’ brain following MIT administration suggests that MIT-modulated mitochondrial dynamics and ER stress could be a result of up-regulation of SIRT1. Indeed, SIRT1 controls the transcription of mitochondrial fission and fusion modulators with the reduction in SIRT1 leading to decreased expression of genes critical for mitochondrial biogenesis and dynamics [28, 74–76].

Likewise, SIRT1 is recognized for its governance over ER stress by influencing the unfolded protein response through key signaling pathways, including the activating transcription factor 6 pathway, which impacts CHOP expression [77, 78]. The SIRT1’s critical role in managing ER stress-induced apoptosis across various cell types has been reported [29, 79, 80].

Interestingly, the results obtained from correlation studies support the link between SIRT1 expression and the expression of mitochondrial dynamics mediators Drp1 and OPA1 and between SIRT1 expression and the expression of CHOP, the ER stress biomarker.

A major consequence of the culminating mitochondrial dysfunction and ER stress is the uncontrolled generation of ROS that overrides the endogenous antioxidant capacity of the cell leading to oxidative stress [81]. Under oxidative stress, the inner mitochondrial membrane becomes more permeable, allowing the release of ions, small molecules, and mtDNA. As a damage-associated molecular pattern, mtDNA can activate an inflammatory immune response [82]. In addition, persistent mitochondrial- and ER-mediated stress activated caspases and suppressed members of the Bcl family, including Bcl-2, thereby eliciting apoptotic cell death [13, 83]. Worth mentioning, the nociceptive system becomes sensitized as a result of neuronal apoptotic alterations [84], and a “wind-up” process is triggered by repeated activation of nociceptors, ultimately aggravating pain sensitization, allodynia, and hyperalgesia [85].

Results obtained from current research revealed provoking of oxidative stress, an inflammatory response and apoptosis in brains of RES-treated rats, which were defined by the significant decrease in SOD and catalase activities associated with a marked upsurge in the NF-κB, TNF-α and BAX brain content, while noticeably diminishing the anti-apoptotic Bcl-2 content. By boosting the antioxidant enzymes and restraining the NF-κB activity, MIT aids in re-establishing the redox imbalance and maintaining cell survival, thus preventing excessive apoptosis and inflammation. This aligns with findings reported by [86–89].

Mounting evidence suggests that SIRT1 impacts multiple biological processes, thereby considerably influencing cellular responses [30]. Current results showed that MIT-induced resistance of RES rats’ brains to oxidative stress, inflammation and apoptosis correlates significantly with an increase in SIRT1 expression. Notably, SIRT1 regulation of ROS has been extensively studied in various tissues with the observation that cells’ lack of sufficient SIRT1 makes them more vulnerable to oxidative stress [90–92]. Also, SIRT1 is capable of regulating the activity of NF-κB, either directly via deacetylation or indirectly through other molecules, thus impacting inflammation and apoptosis [35, 93, 94].

A key discovery from the current research is that MIT significantly decreased the genetic expression of miRNA-320 in the brains of rats affected by RES, with an inverse relation was detected between miRNA-320 expression and the expression of SIRT1. As previously noted, alterations in patterns of different miRNAs expression were noticed in FM patients [95]. In particular, up-regulated miRNA-320 was noticed in FM patients who also had headache, depression and generalized fatigue [22]. In biopsies taken from patients having chronic bladder pain syndrome, Freire and coworkers have reported that the upregulated miRNA-320a induced a statistically significant decrease in neurokinin 1 receptor protein levels, hence implicating the process of pain transmission [96].

Remarkably, the inverse relation detected between miRNA-320 and SIRT1 expression has acknowledged the regulatory control exerted by each on the other and harmonized with that stated by previous studies [35, 97–99].

## Conclusion

In summary, the results from this study highlight the extensive influence of MIT on various molecular pathways related to FM. By activating SIRT1, MIT could adjust mitochondrial dynamics, reduce mitochondrial- and ER-mediated oxidative stress and apoptosis and exert epigenetic regulation on miRNA-320. Together these mechanisms significantly rebalanced the brain levels of biogenic amines to improve pain perception and lessen depressive-like behaviors in rats with FM.

Though current findings are critical therapeutic milestones, emphasizing the significance of considering MIT as a potential intervention in FM, they are preliminary. Additional studies are required to select the maximum effective dose, fully clarify the protective mechanism of MIT in FM-like model, and explore MIT’s potential clinical benefits and safety for treating FM.

## Electronic Supplementary Material

Below is the link to the electronic supplementary material.

Supplementary Material 1

Supplementary Material 2

Supplementary Material 3

## Data Availability

No datasets were generated or analysed during the current study.

## Abbreviations

5-HT: 5-hydroxytryptamine
ANOVA: Analysis of variance
ATP: Adenosine triphosphate
BAX: Bcl-2 associated X protein
Bcl-2: B cell lymphoma
CAT: Catalase
CHOP: C/EBP homologous protein
DA: Dopamine
DRP1: Dynamin-related protein 1
eIF2α: Eukaryotic translation Initiation Factor 2 alpha
ELISA: Enzyme-linked immunosorbent assay
ER: Endoplasmic reticulum
FM: Fibromyalgia
FOXO: Forkhead box O protein
FST: Forced swimming test
i.p.: Intraperitoneal
miRNA: micro RNA
MIT: Mito-TEMPO
mtDNA: Mitochondrial DNA
NAD: Nicotinamide adenine dinucleotide
NE: Norepinephrine
NF-κB: Nuclear factor kappa B
OFT: Open field test
OPA1: Optic atrophy factor 1
P 53: Tumor protein P53
P38 MAPK: p38 mitogen-activated protein kinases
PERK: Protein kinase RNA-like endoplasmic reticulum kinase
PG: Pregabalin
r: Correlation coefficient
RES: Reserpine
ROS: Reactive oxygen species
RT-qPCR: Real-time quantitative polymerase chain reaction
s.c.: Subcutaneously
SD: Standard deviation
SIRT 1: Silent information regulator 1
SOD: Superoxide dismutase
TNF-α: Tumor necrosis factor -α
