# Supplementary figures and images for: Mito-TEMPO Mitigates Fibromyalgia Induced by Reserpine in Rats: Orchestration Between SIRT1, Mitochondrial Dynamics, Endoplasmic Reticulum and miRNA-320
Source: Neurochem Res. 2025 May 28;50(3):172. doi: 10.1007/s11064-025-04424-9 (PMC12119751; doi:10.1007/s11064-025-04424-9)

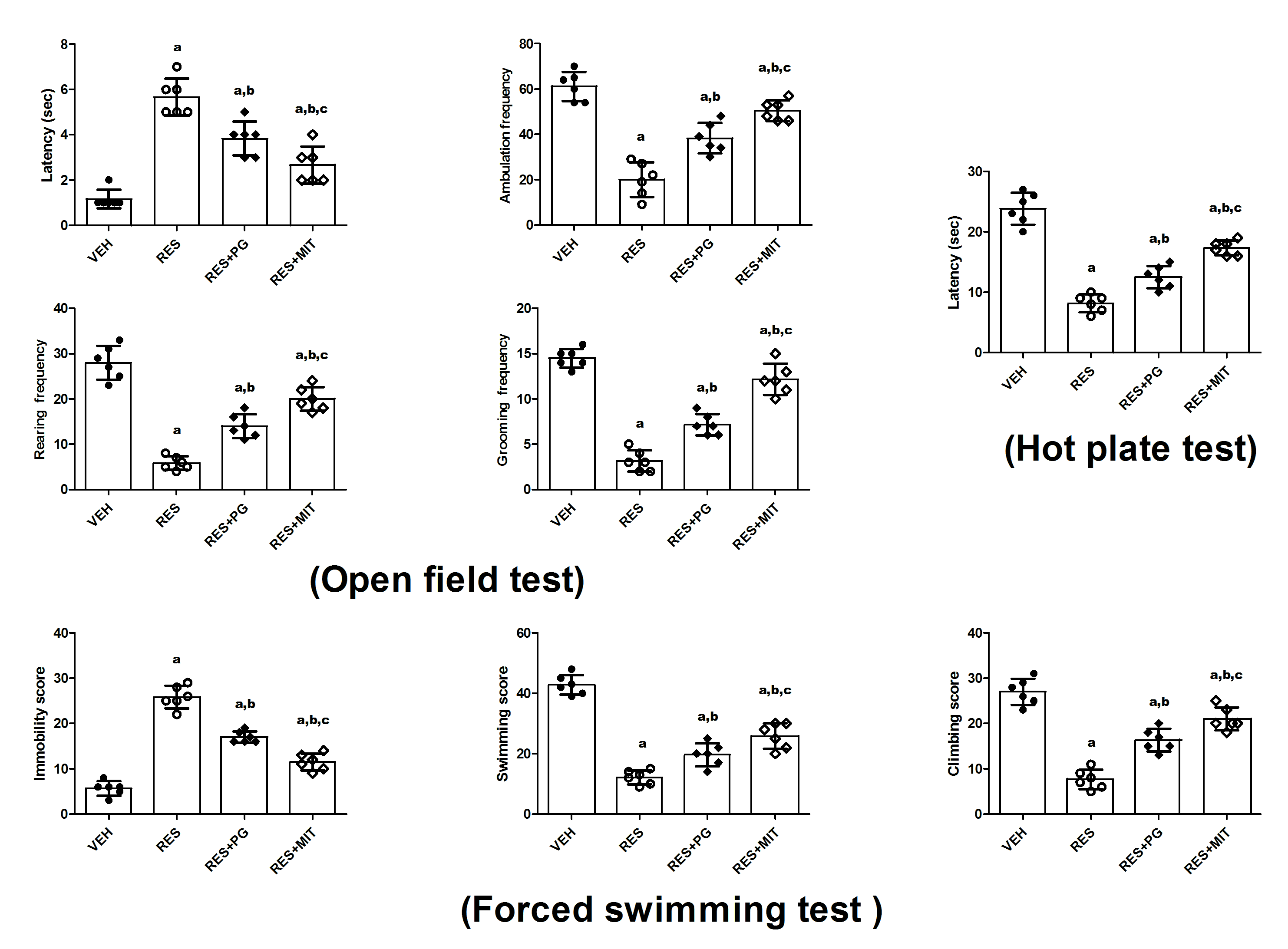

Supplement: Supplementary file 1 — Supplementary Material 1 [file 11064_2025_4424_MOESM1_ESM.tif]

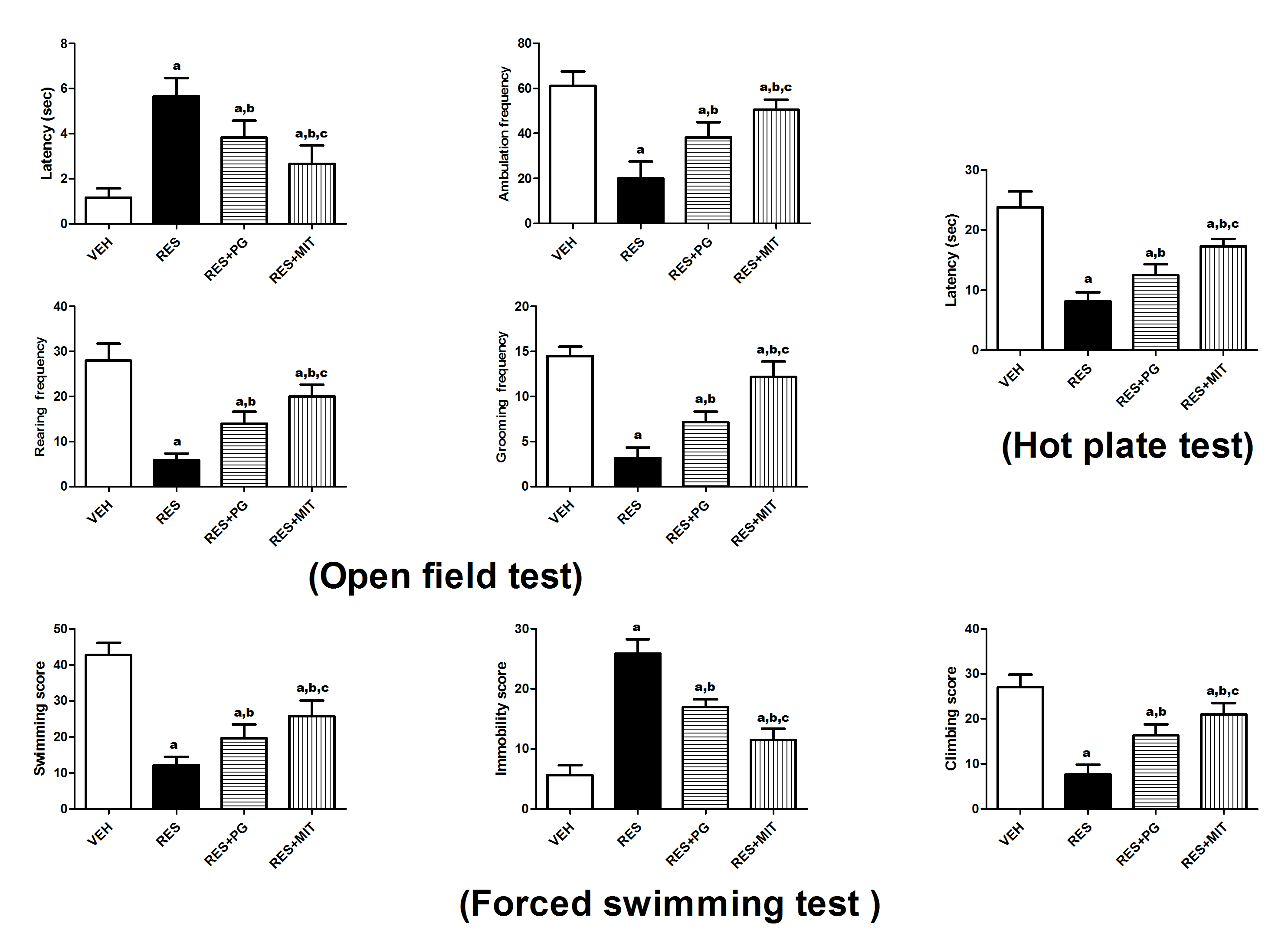

Supplement: Supplementary file 2 — Supplementary Material 2 [file 11064_2025_4424_MOESM2_ESM.tif]
