# Supplementary material for: Mito-TEMPO Mitigates Fibromyalgia Induced by Reserpine in Rats: Orchestration Between SIRT1, Mitochondrial Dynamics, Endoplasmic Reticulum and miRNA-320
Source: Neurochem Res. 2025 May 28;50(3):172. doi: 10.1007/s11064-025-04424-9 (PMC12119751; doi:10.1007/s11064-025-04424-9)
Supplement: Supplementary file 3 — Supplementary Material 3 [file 11064_2025_4424_MOESM3_ESM.docx]

**Supplementary figures legends**

**Fig. 1S: Effect of Mito-TEMPO on fibromyalgia-induced changes in biogenic amines in rats’ brain**

Values explicate the mean ± SD (n=6). Statistics were carried out by ANOVA tailed by Tukey’s as post-hoc test. **^a^,** **^b^, ^c^** statistically significant from VEH, RES and RES+PG groups respectively at **p < 0.05. ANOVA**: analysis of variance; **5-HT**: 5-hydroxytryptamine; **VEH**: Vehicle group; **DA**: Dopamine; **RES**: Fibromyalgia model group; **RES+MIT**: Mito-TEMPO-treated fibromyalgia group; **RES+PG**: pregabalin-treated fibromyalgia group; **NE**: Norepinephrine; **SD**: Standard deviation.

**Fig. 2S: Effect of Mito-TEMPO on fibromyalgia-induced behavioral changes in rats**

Values explicate the mean ± SD (n=6). Statistics were carried out by ANOVA tailed by Tukey’s as post-hoc test. **^a^,** **^b^, ^c^** statistically significant from VEH, RES and RES+PG groups respectively at **p < 0.05. ANOVA**: analysis of variance; **VEH**: Vehicle group; **RES**: Fibromyalgia model group; **RES+MIT**: Mito-TEMPO -treated fibromyalgia group; **RES+PG**: Pregabalin-treated fibromyalgia group; **no**: Number; **SD**: Standard deviation; **sec**: Seconds.
